# Protein and Signaling Networks in Vertebrate Photoreceptor Cells

**DOI:** 10.3389/fnmol.2015.00067

**Published:** 2015-11-17

**Authors:** Karl-Wilhelm Koch, Daniele Dell’Orco

**Affiliations:** ^1^Department of Neurosciences, Biochemistry Group, University of OldenburgOldenburg, Germany; ^2^Department of Neurological, Biomedical and Movement Sciences, Section of Biological Chemistry and Center for BioMedical Computing (CBMC), University of VeronaVerona, Italy

**Keywords:** multi-protein complexes, second messenger signaling, phototransduction, cGMP, calcium-binding proteins

## Abstract

Vertebrate photoreceptor cells are exquisite light detectors operating under very dim and bright illumination. The photoexcitation and adaptation machinery in photoreceptor cells consists of protein complexes that can form highly ordered supramolecular structures and control the homeostasis and mutual dependence of the secondary messengers cyclic guanosine monophosphate (cGMP) and Ca^2+^. The visual pigment in rod photoreceptors, the G protein-coupled receptor rhodopsin is organized in tracks of dimers thereby providing a signaling platform for the dynamic scaffolding of the G protein transducin. Illuminated rhodopsin is turned off by phosphorylation catalyzed by rhodopsin kinase (GRK1) under control of Ca^2+^-recoverin. The GRK1 protein complex partly assembles in lipid raft structures, where shutting off rhodopsin seems to be more effective. Re-synthesis of cGMP is another crucial step in the recovery of the photoresponse after illumination. It is catalyzed by membrane bound sensory guanylate cyclases (GCs) and is regulated by specific neuronal Ca^2+^-sensor proteins called guanylate cyclase-activating proteins (GCAPs). At least one GC (ROS-GC1) was shown to be part of a multiprotein complex having strong interactions with the cytoskeleton and being controlled in a multimodal Ca^2+^-dependent fashion. The final target of the cGMP signaling cascade is a cyclic nucleotide-gated (CNG) channel that is a hetero-oligomeric protein located in the plasma membrane and interacting with accessory proteins in highly organized microdomains. We summarize results and interpretations of findings related to the inhomogeneous organization of signaling units in photoreceptor outer segments.

## Introduction

**Figure 1:**
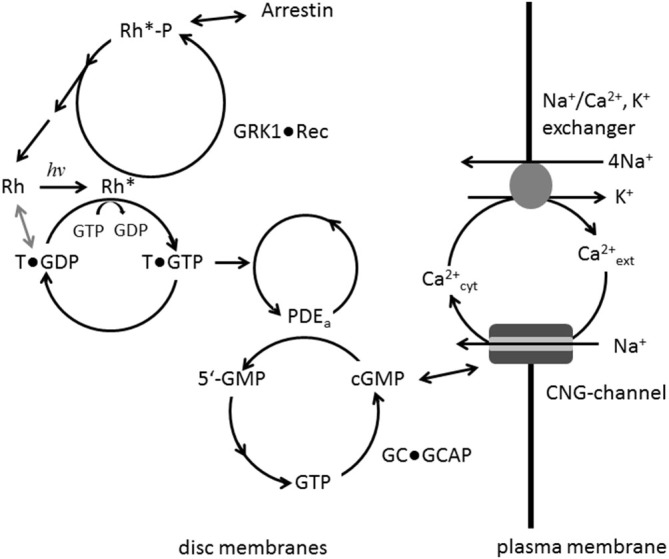
**Main signaling steps in phototransduction.** Photo-activation of rhodopsin (Rh to Rh*) leads to GDP/GTP exchange at the G protein transducin (T) which in turn activates its effector PDE. Hydrolysis of cGMP is catalyzed by activated PDE; resynthesis of cGMP by guanylate cyclase (GC) is under control of a negative Ca^2+^-feedback involving the GC-activating proteins (GCAP) Ca^2+^-sensor proteins. Ca^2+^ enters the cell via the cyclic nucleotide-gated (CNG)-channel and is extruded by the exchanger. Rh* is phosphorylated by GRK1, when inhibition by Ca^2+^-bound recoverin is relieved. Arrestin can bind to phosphorylated Rh* preventing further activation of transducin.

Vertebrate photoreceptor cells are neurosensory cells of unique morphology and specialized function. They are divided into two general types, rods and cones, which mediate vision at night and daylight, respectively. Absorption of photons by visual pigments, rhodopsin in rods and cone opsins in cones, triggers a well understood signaling cascade that has been thoroughly investigated in the past decades. Numerous articles in the last decades have therefore summarized the basic features of the phototransduction process (e.g., Stryer, 1991; Kaupp and Koch, 1992; Koch, 1994; Pugh and Lamb, 2000; Luo et al., 2008; Wensel, 2008; Arshavsky and Burns, 2012; Korenbrot, 2012; Palczewski, 2012): coupling of visual pigments to the heterotrimeric G protein transducin, activation of the effector phosphodiesterase PDE6 by the G protein and efficient hydrolysis of the second messenger cyclic nucleotide guanosine 3^′^,5^′^-cyclic monophosphate (cGMP) with high turnover rates, regulation of the cyclic nucleotide-gated (CNG)-channel by cGMP and re-synthesis of cGMP by a guanylate cyclase (GC) complex that is controlled by a powerful Ca^2+^-dependent feedback loop (Dizhoor et al., 2010; Koch et al., 2010). The cytoplasmic Ca^2+^-concentration in the outer segments of photoreceptor cells is maintained by two transport routes: Ca^2+^-influx through the CNG-channel and Ca^2+^-extrusion by Na^+/^Ca^2+^, K^+^-exchanger. Due to the light-dependent closure of the CNG-channel (Kaupp and Seifert, 2002), Ca^2+^ cannot enter the cell, but is expelled via the exchanger leading to a net decrease in cytoplasmic Ca^2+^. Termination of the signaling cascade is an equally critical step for the precise formation of light responses and photoresponse recovery. All activation steps of the excitation pathway need efficient shut-off mechanisms (Burns, 2010). Thus, rhodopsin is phosphorylated by rhodopsin kinase (GRK1) under control of Ca^2+^/recoverin (Senin et al., 2002b) and competitive binding of arrestin to phospho-rhodopsin prevents further interaction of transducin with rhodopsin (Sommer et al., 2014). As regards the effector, the acceleration of the intrinsic GTPase activity of transducin by RGS9-1 and accessory proteins Gβ5L and R9AP leads to a sub-second deactivation of transducin (Wensel, 2008), which in turns dissociates from PDE6, thus restoring the low basal activity controlled by its small inhibitory γ subunits. Figure 1 summarizes the basic features of the phototransduction cascade.

The detailed knowledge about the physiology and biochemistry of phototransduction has been covered in numerous in-depth reviews (for a small selection see references above). The present article, however will address issues, which had been investigated in more recent years showing that the photoreceptor outer segment is not a well-stirred compartment, but appears rather inhomogeneous. Publications indicating different aspects of heterogeneity date back several decades, but have been mainly interpreted as signs of the natural ageing and renewal process in outer segments. For example, gradients along the longitudinal axis of outer segments have been described for second messenger molecules (Leibovic and Bandarchi, 1997a,b; Gray-Keller et al., 1999), disc membrane composition (Boesze-Battaglia et al., 1989, 1990; Boesze-Battaglia and Albert, 1990), light response variation (Baylor and Lamb, 1982; Schnapf, 1983; Mazzolini et al., 2015) and enzymatic reactions (Shichi and Williams, 1979). While the ageing process is out of question the physiological consequences of gradients of intracellular components and the heterogeneous formation of multi-protein units come into focus. Proteins of the photoexcitation and adaptation machinery are assembled in complexes that can form highly ordered supramolecular structures by interacting with the supporting disc membrane vesicles (Wensel, 2008). Moreover, some of the main molecular components of the phototransduction cascade have been found to be involved in other non-visual related signaling networks (Kiel et al., 2011). Thus, we will discuss in the present review how these signal transducing modules contribute to the efficient and precise processing of light signals. We have restricted our review to rod biochemistry and physiology, since knowledge about cones and about protein and signaling networks in cones is less advanced. Moreover, it is worth noting that some aspects concerning for example *the supramolecular organization of rhodopsin* (see below) have not been investigated yet for the cone system so far.

## Photoresponse Gradient Along the Longitudinal Axis of Photoreceptor Outer Segments

Suction electrode recordings from amphibian rods showed already in the 1980s that single flash response kinetics are slower when the cell is illuminated at the tip of the rod outer segment than at the base. Response amplitudes become smaller the farther away from the base the flash is delivered (Schnapf, 1983). Outer segments are renewed every 10 days in mammalians and 6–7 weeks in amphibians by a process called disc shedding, in which the disc components at the tip of the outer segments are phagocytized by the retinal pigment epithelium (Young, 1967). This process keeps the length of the outer segment constant under physiological conditions and the heterogeneity of flash response kinetics and amplitudes were interpreted as the results of an ageing process. Mazzolini et al. (2015) have recently confirmed and extended these earlier observations using dissociated rods from adult male *Xenopus laevis* frogs. However, their analysis showed that the amplitude of saturating and single photon responses decreased by 5–10 times, when illumination of the tip is compared with that of the base. Previous recordings showed only a twofold difference in sensitivity. Mazzolini et al. (2015) excluded a lower probability of photon capture by rhodopsin, but instead suggested that a reduced amplification of the transduction cascade leads to a reduction in efficacy due to a progressive depletion of PDE6 along the longitudinal axis of rod outer segment. Further, the kinetic variability of the light responses at the base, middle and tip of the outer segment is not contemplated by the existing quantitative models, which operate under the assumption that the biochemical components of the transduction machinery are more or less uniformly distributed (Hamer et al., 2005; Bisegna et al., 2008; Dell’Orco et al., 2009; Shen et al., 2010). Instead, the authors suggest a “series of interconnected compartments” that might have different local concentrations of key factors and therefore vary in their responsiveness to light.

## Supramolecular Organization of Rhodopsin

Efficient photon capture by photoreceptor cells is due to the high density of the visual pigment rhodopsin in stacks of disc membranes reaching ≥25,000 μm^-2^. Work from the early 1970s came to the conclusion that rhodopsin is homogenously distributed in disc membranes and can laterally diffuse without much restriction (Cone, 1972; Liebman and Entine, 1974; Poo and Cone, 1974). This concept of random distribution and free diffusion in the membrane was further supported by results obtained by neutron diffraction and electron microscopy (Saibil et al., 1976; Roof and Heuser, 1982). After being accepted in the field for 30 years this “classical” view was challenged by an atomic force microscopy (AFM) study of disc membranes, which showed in 2003 that rhodopsin dimers are found in a paracrystalline arrangement suggesting a lower degree of lateral diffusion (Fotiadis et al., 2003a, 2004). Subsequent studies were in agreement with a more inhomogeneous distribution of immobile rhodopsin fractions (Govardovskii et al., 2009). Another very recent independent AFM study revealed that dimers of rhodopsin are organized in nanodomains, whose supramolecular features seem to be conserved in humans and mice. For example, the discs diameter was found to depend on the number of rhodopsin molecules embedded in the membrane, however it was found to be independent of rhodopsin’s spatial density (Whited and Park, 2015). A different study further reported highly concentrated patches of rhodopsin in the central region of a disc. No rhodopsin was located near the rim region that was occupied by peripherin and Rom proteins, which are well established marker proteins for this region of the outer segment (Buzhynskyy et al., 2011).

Very recently, using cryosections of dark-adapted intact rod photoreceptors from mice Gunkel et al. (2015) demonstrated by cryoelectron tomography the presence of tracks of rhodopsin dimers. In a scenario only partly similar to previous AFM determinations, the authors observed that at least ten rhodopsin dimers form a row, rows form pairs (tracks), and tracks are aligned parallel to the disc incisures, with profound implications for the kinetics of phototransduction.

Soon after the classical view of randomly distributed and freely diffusing rhodopsin monomers was challenged, a controversial discussion on this topic started and had not ended so far (Chabre et al., 2003; and reply by Fotiadis et al., 2003b). Previous biochemical and biophysical studies demonstrated that monomeric rhodopsin is sufficient to activate transducin, leading to the conclusion that no mechanistic need for a rhodopsin dimer exists (Chabre and le Maire, 2005; Ernst et al., 2007). Biochemical studies of detergent solubilized rhodopsin however provided evidence for the existence of rhodopsin dimers or oligomers depending on the detergent and solubilization conditions (Jastrzebska et al., 2004). But the use of detergents is prone to affect the quaternary structure of proteins and was therefore considered as a weak argument to support the existence of higher order organization rhodopsin in disc membranes (Chabre and le Marie, 2004). The paracrystalline arrangement of rhodopsin dimers observed in the early AFM studies was interpreted as the results of separation of the lipid phase from proteins at low temperatures (Chabre et al., 2003), but the higher order topography was also observed at room temperature (Fotiadis et al., 2003b), moreover recent AFM determinations confirmed that higher order nano-domains of rhodopsin are found both in mice and humans (Whited and Park, 2015).

A very recent study investigated the nature of rhodopsin dimers by showing that peptides encompassing the transmembrane domains (TMs) of rhodopsin block its dimerization both *in vitro* and in living cells, without however affecting the rates of transducin activation (Jastrzebska et al., 2015). This suggests that the supramolecular organization of rhodopsin could be essential for the stabilization of rod outer segments and receptor trafficking rather than for activating the G protein.

## Dynamics of Transient Protein Complexes Involving Rhodopsin

**Figure 2:**
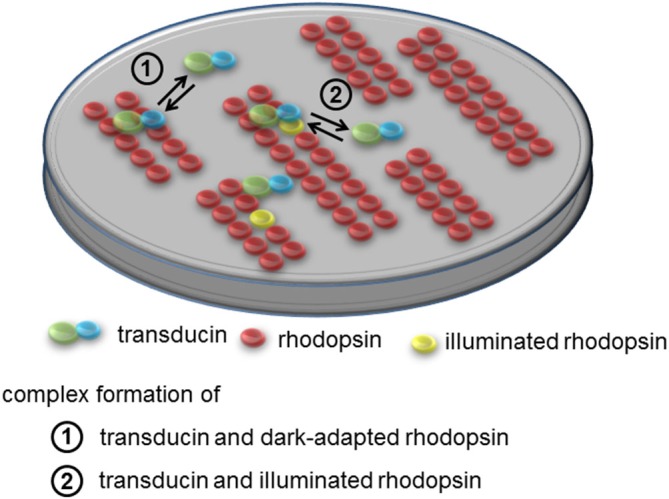
**Supramolecular organization of rhodopsin and interaction with transducin.** Rhodopsin is present in tracks of dimers in the disc membrane. In the dark rhodopsin-transducin complexes form with submicromolar affinity that is characterized by very fast association and dissociation rates. Movements of transducin can be described as dynamic hopping on rhodopsin supramolecular assemblies thus constituting “dynamic scaffolding”. Apparent dissociation rates of transducin from dark-adapted rhodopsin are >300-fold faster than corresponding rates from light-activated rhodopsin.

The supramolecular organization of nearly immobile rhodopsin imposes a conceptual problem on the mechanistic understanding of the phototransduction cascade, which operates on a millisecond time base with high sensitivity. Intuitively one might predict that arrays of paracrystalline rhodopsin would slow down the activation kinetics. The investigation of how the diffusion properties of rhodopsin and transducin are affected by specific supramolecular assemblies is not trivial, and has so far been investigated only by computational analyses that incorporate available experimental data. Results were not always intuitive, because rhodopsin supramolecular assemblies and molecular crowding in discs imply anomalous and anisotropic diffusion paths for peripheral membrane proteins such as transducin, which significantly differ from the classical free diffusion case. In a first approach to the problem, Monte Carlo simulations of rhodopsin-transducin encounters in both the classical free diffusion and emerging paracrystalline rafts scenarios suggested that an unexpected favorable effect on the temporal response of early phototransduction reactions may occur, if rhodopsin molecules were packed in highly ordered assemblies (Dell’Orco and Schmidt, 2008). Moreover, recent surface plasmon resonance studies (Dell’Orco and Koch, 2011) performed with detergent solubilized native rhodopsin demonstrated the existence of a protein-protein interaction between dark-adapted rhodopsin and transducin, which was postulated earlier based on the analysis of their structural complementarity (Fanelli and Dell’Orco, 2005; Dell’Orco et al., 2007). Dark rhodopsin-transducin binding occurs with submicromolar affinity and is characterized by very fast association and dissociation rates in a “dynamic scaffolding” frame, where concerted diffusion/binding phenomena give rise to a dynamic hopping of transducin on rhodopsin supramolecular assemblies (Dell’Orco and Koch, 2011). The transient precoupling step was integrated into the framework of phototransduction models of both amphibian and murine rods, and was found to be compatible with the overall cascade kinetics (Invergo et al., 2014; Dell’Orco, 2015). The physiological presence of rhodopsin-transducin transient complexes has been somewhat questioned and debated (Schöneberg et al., 2014, 2015; Dell’Orco and Koch, 2015), however it appears now quite clear that it may have deep implications for the capability of rods to detect single photons (Cangiano and Dell’Orco, 2013; Dell’Orco, 2013), and seems to be an essential mechanistic step in the recently emerged picture of rhodopsin tracks observed by cryoelectron tomography, in order to create the “kinetic traps”: owing to the frequent, rapid formation and breakup of precomplexes, transducin molecules could scan a rhodopsin track by discrete hopping events, resulting in an activation rate that, in the single-photon regime, would be determined by the number of the preassembled transducin molecules per track rather than the photoactivated rhodopsin lifetime. The number of transducin molecules activated per photoactivated rhodopsin would be therefore of the same order as the number of preassembled transducin molecules per unit track (Gunkel et al., 2015). The concepts developed in the last years in opposition to the “fluid mosaic” classical organization of the disc membrane have built up a novel structural picture of the early mechanisms triggering phototransduction, whose supramolecular features are summarized in Figure 2.

Proteomic profiling and protein network analysis of outer segments led to the prediction of signaling and/or trafficking pathways in addition to the activation and deactivation pathways that govern photoreceptor excitation and recovery. An important level of regulation of such alternative pathways seems to be played by small GTPases (Kiel et al., 2011). The monomeric G-protein Rac1 is among the putative binding partners of rhodopsin (Balasubramanian and Slepak, 2003), but its lower cellular concentration (~100-fold excess of rhodopsin) and its medium affinity for rhodopsin (apparent K_D_ = 1.3 μM) would not allow a significant competition with the binding of transducin (Köster et al., 2014). However, under strong illumination, when transducin is depleted from the outer segment by transport to the inner segment (Pulvermüller et al., 2002; Sokolov et al., 2002; Lobanova et al., 2007), only about 10% of all rhodopsin molecules could form a complex with Rac1. Therefore, it is more likely that Rac1 binds to rhodopsin during transport after protein biosynthesis. Intracellular trafficking, in particular under conditions of changing illumination has attained increasing interest in the photoreceptor research community. In order to keep this review focused we will not discuss this field in depth, but will refer to some reviews on this topic (Calvert et al., 2006; Karan et al., 2010; Pearring et al., 2013; Wang and Deretic, 2014).

## Deactivation of Rhodopsin

The efficient shut-off of the phototransduction cascade requires as initial step the deactivation of photoactivated rhodopsin. This crucial step is performed by the interplay of several proteins and binding events: GRK-1 phosphorylates illuminated rhodopsin at its C-terminus (Maeda et al., 2003), which allows subsequent binding of arrestin (p48) or the arrestin splice variant p44 (Granzin et al., 2012; Kim et al., 2013). Binding of arrestin to phosphorylated rhodopsin prevents further activation of transducin (Pulvermüller et al., 1993).

**Figure 3:**
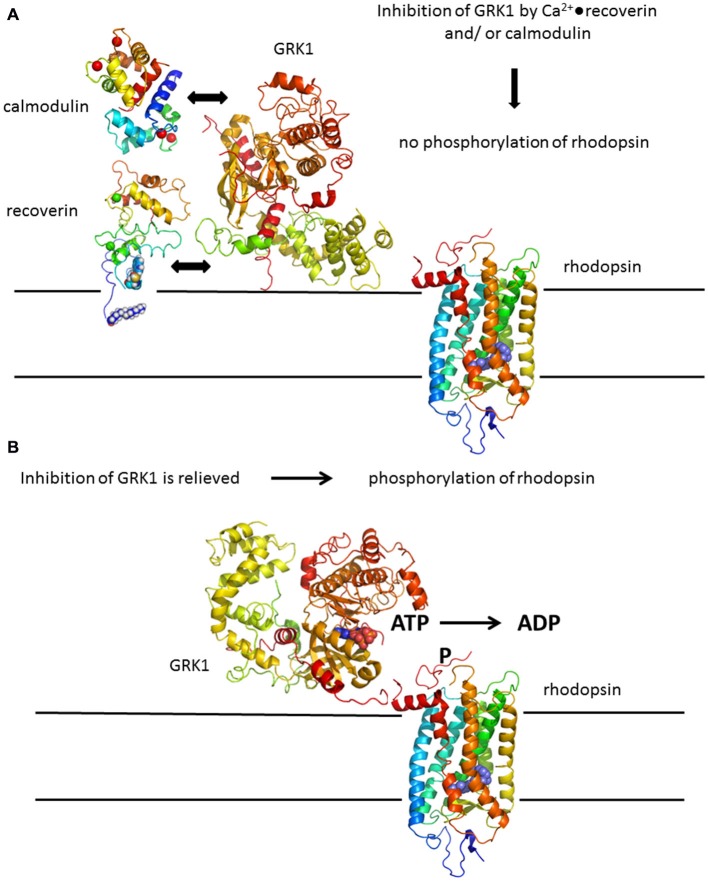
**Rhodopsin phosphorylation by GRK1. (A)** Deactivation of rhodopsin is under control of a Ca^2+^-feedback loop involving Ca^2+^-sensor proteins recoverin and calmodulin. Both Ca^2+^-binding proteins have non-overlapping binding sites in GRK1 and act in a synergetic mode, for example by increasing the Ca^2+^-sensitivity of GRK1 regulation. **(B)** Inhibition of GRK1 is relieved at decreasing Ca^2+^-concentration after illumination leading to phosphorylation of rhodopsin. Structures were prepared with pymol; corresponding PDB codes are: 1F88 for rhodopsin (Palczewski et al., 2000); 3C51 for GRK1 (Singh et al., 2008); 1JSA for recoverin (Ames et al., 1997); 1CDM for calmodulin (Meador et al., 1993).

Serine and threonine residues present in the C-terminus of rhodopsin within the amino acid positions 324–348 are potential sites for phosphorylation by GRK1. Like other members of the GRK family, GRK1 phosphorylates only the light-stimulated (bleached) form of the receptor and does not act upon the unbleached receptor (for reviews, see Senin et al., 2002b; Maeda et al., 2003; Premont and Gainetdinov, 2007). Activity of GRK1 is under control of a Ca^2+^-dependent negative feedback loop, but GRK1 itself is not sensitive to Ca^2+^. Changes in intracellular [Ca^2+^] are sensed by retina specific neuronal Ca^2+^-sensor proteins including the Ca^2+^-binding protein recoverin (Senin et al., 2002b). By interacting with GRK1 in a Ca^2+^-dependent manner it controls GRK1 enzyme activity (Kawamura, 1993; Gorodovikova et al., 1994; Klenchin et al., 1995). Recoverin is heterogeneously acylated at its N-terminus by an N-myristoyl-transferase like activity (Dizhoor et al., 1992). When acylated recombinant forms of recoverin are investigated, a myristoyl group is routinely attached to the protein by co-expression of a N-myristoyl-transferase. The myristoyl moiety has a strong impact on the structural and functional properties of recoverin. For example, in the absence of Ca^2+^, the myristoyl group is buried within a hydrophobic pocket of the protein (Tanaka et al., 1995). Binding of Ca^2+^ to recoverin triggers a conformational change that leads to exposure of the myristoyl group and the hydrophobic pocket (Zozulya and Stryer, 1992; Ames et al., 1997), which facilitates its association with biological membranes and inhibition of GRK1 (Chen et al., 1995; Senin et al., 1995). Since the cytoplasmic Ca^2+^-concentration is high in the resting dark state of the photoreceptor cell, Ca^2+^-loaded recoverin associates with the disc membranes, interacts with GRK1 and inhibits its activity. Decrease of intracellular Ca^2+^ after illumination triggers dissociation of recoverin from the membranes and from GRK1 thereby stopping the inhibition of GRK1 and allowing rhodopsin phosphorylation (Figures 3A,B).

The Ca^2+^-myristoyl switch of recoverin (Zozulya and Stryer, 1992) is prototypical for a variety of myristoylated proteins and has served as a benchmark for studying related switching mechanisms in other Ca^2+^-sensors (O‘Callaghan et al., 2003; Li et al., 2011; Lim et al., 2011, 2014; Burgoyne and Haynes, 2015; Marino et al., 2015; Sulmann et al., 2015) or by characterizing the biocompatibility of nanoparticles (Marino et al., 2014). The Ca^2+^-binding and switching process in recoverin had been investigated in more detail by comparing WT recoverin with mutants containing disabled EF-hands or a truncated C-terminus (Senin et al., 2002a, 2003; Weiergräber et al., 2003, 2006). These studies revealed critical steps in sequential Ca^2+^-binding and defined crucial regions that control the Ca^2+^-sensitive regulation of GRK1. Several studies were also focused on regulatory aspects of GRK1 using site-directed mutagenesis, pull down methods, SPR spectroscopy and rhodopsin phosphorylation assays (Huang et al., 2009, 2011; Komolov et al., 2009; Zernii et al., 2011; Orban et al., 2012). The N-terminus of GRK1 forms an amphipathic α–helix, of which the first 25 amino acid residues interact with an exposed hydrophobic groove in recoverin (Ames et al., 2006; Higgins et al., 2006). A combination of structural analysis and computational modeling of the recoverin-kinase complex revealed that the protein-protein interface involves also the C-terminus of recoverin by forming a cation-π interaction pair, which is essential for GRK1 target recognition by recoverin (Zernii et al., 2011). Fine-tuning of Ca^2+^-dependent regulation of GRK1 is further achieved by synergetic action of calmodulin (Figure 3A), which binds to a region in GRK1 distant from the recoverin binding site (Grigoriev et al., 2012).

Recent electrophysiological recordings on transgenic mice lacking recoverin (Rv^−/−^) showed shortened light responses indicating a reduced lifetime of light-activated rhodopsin (Makino et al., 2004; Bush and Makino, 2006; Chen et al., 2010), which is consistent with an inhibition of GRK1 by recoverin at high [Ca^2+^]. The same effect was recently observed by intra ocular and ex-vivo retinal delivery of liposomes loaded with recombinant recoverin or its antibody, which showed effects comparable to recoverin overexpression and downregulation, respectively (Asteriti et al., 2015). Similarly, overexpression of GRK1 in transgenic mice (12-fold higher than in wildtype (WT) mice) also leads to shortened light responses (Chen et al., 2012). By investigating mice harboring different genetic manipulations Chen et al. (2012) challenged previous investigations on mice expressing GRK1 at lower and higher levels than WT (Sakurai et al., 2011) and postulated a time constant of rhodopsin deactivation of about 50 ms. In a different approach using bottom-up modeling, the rate limiting steps in the recovery of rods after illumination were investigated by simulating conditions, in which the expression levels of GRK1 and recoverin were altered individually or in combination (Invergo et al., 2013). The analysis provided a mechanistic explanation for the puzzling evidence that GRK1 over expression does not influence the saturation time of rods under bright light stimulation (Krispel et al., 2006; Sakurai et al., 2011), indeed attributing a compensating effect to arrestin oligomerization (Invergo et al., 2013). The recoverin-GRK1 complex in cones is operating under dim light, but not in bright light demonstrating significant differences in the response recovery between rod and cone physiology (Sakurai et al., 2015). Very recently, Chen et al. (2015) suggested that GRK1 and recoverin participate in the activity regulation of PDE6, but biochemical evidence to support this hypothesis is lacking so far.

## Signaling Modules and Lipid Rafts

Patches of rhodopsin seen in AFM images (see above) were interpreted as lipid raft structures and biochemical fractionation studies pointed to the existence of lipid rafts in rod outer segments. Detergent resistant membranes or lipid rafts can be isolated from sucrose step gradients of cell suspensions that are solubilized with a low concentration of the nonionic detergent Triton X-100. Main features of lipid rafts are high cholesterol content, saturated fatty acids, glycolipids and the marker protein caveolin (Martin et al., 2005; Elliott et al., 2008) that was shown to co-localize with signaling proteins like transducin in rod outer segments (Elliott et al., 2003). In fact, whole signaling units have been identified in isolated lipid rafts from preparations of rod outer segments. For example, rhodopsin, transducin, its effector cGMP-phosphodiesterase, the shorter splice variant of arrestin p44 and the RGS9-Gβ5L complex translocate to raft structures in a light-dependent manner (Seno et al., 2001; Nair et al., 2002; Balasubramanian and Slepak, 2003; Liu et al., 2003). Main conclusions from these studies were that the rhodopsin-transducin coupling is reduced and that phototransduction is less efficient in lipid micro-domains. Raft-specific protein complexes including rhodopsin were also suggested to have a role in the formation of outer segments (Berta et al., 2011).

Interestingly, relative protein composition of the raft fraction from rod outer segments was not only dependent on illumination, but also on the free Ca^2+^-concentration (Senin et al., 2004). Changing free Ca^2+^ causes proteins to translocate between the soluble fraction and the detergent resistant membrane fraction. Manipulation of the cholesterol content by the reagent methyl-β-cyclodextrin showed a clear dependence of recoverin controlled GRK1 activity: the Ca^2+^-dependent activity profile of GRK1 is shifted to higher Ca^2+^, when cholesterol is low and to lower Ca^2+^, when cholesterol is high (Senin et al., 2004). In native cells cholesterol is not homogeneously distributed in outer segment disc membranes, but instead rod outer segments contain a cholesterol gradient (high to low) from the base to the tip (Boesze-Battaglia et al., 1989, 1990; Boesze-Battaglia and Albert, 1990). This means, in terms of the cell’s physiology, that rhodopsin shut-off by phosphorylation would be more effective at the base than at the tip leading to faster deactivation kinetics at the base. It remains an open question, whether the photoresponse gradient along the longitudinal axis (see above) results from a lipid raft gradient. If this were the case, the photoresponse gradient would in fact depend on the local supramolecular assembly of phototransduction components.

## GC Protein Complex

**Figure 4:**
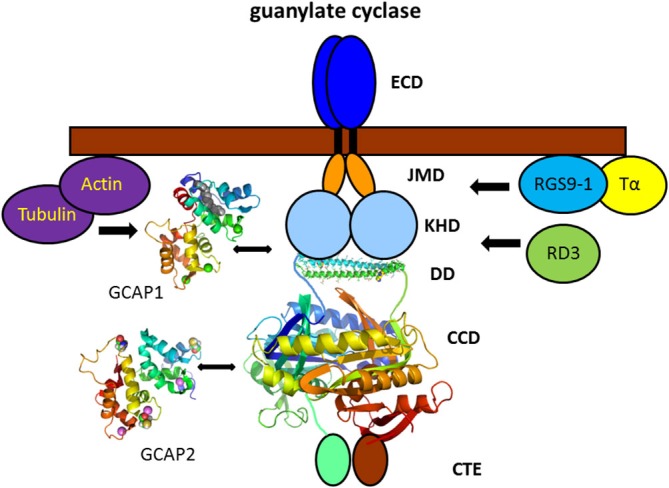
**Vertebrate photoreceptor GC and interacting proteins.** Photoreceptor GCs contain several domains denoted as extracellular domain (ECD, which in rod outer segment is present in the intradiscal lumen; TM, transmembrane domain; JMD, juxtamembrane domain; KHD, kinase homology domain; DD, dimerization domain; CCD, cyclase catalytic domain; and CTE, a C-terminal extension). The tertiary structure of the DD and CCD is adapted from the solved three-dimensional structure of soluble GCs (Ma et al., 2010; Allerston et al., 2013) that display a high sequence homology with membrane bound GCs in these domains (PDB codes: 3HLS and 3UVJ). Assembly and topography of GC domains, in particular of the DD and CCD is arbitrarily chosen. GCAP1 and GCAP2 activate the target GC at low cytoplasmic Ca^2+^-concentration bringing the cell back to the dark state. The exact regions of interaction and/or regulation by GCAPs are a matter of debate (see main text). Interaction with other proteins was shown by biochemical procedures, but physiological meaning is lacking so far. Structures of GCAP1 and GCAP2 are based on the published x-ray and NMR-structure, respectively (GCAP1: 2R2I, Stephen et al., 2007; GCAP2: 1JBA, Ames et al., 1999).

In addition to signaling proteins that translocate to raft structures in a light—dependent manner, the membrane bound GC in photoreceptor cells is associated with rafts independent of the illumination conditions. This membrane bound sensory GC is expressed in two forms in vertebrate rod and cone cells (Dizhoor et al., 1994; Goraczniak et al., 1994; Lowe et al., 1995) and association of one form, presumably ROS-GC1 (synonymously named GC-E or RetGC1), had been detected by immunoblotting in detergent resistant membrane fractions (Nair et al., 2002; Senin et al., 2004). It is known, however from previous studies that GC activities co-fractionate with axonemes (Fleischmann and Denisevich, 1979) of rod cells and purification of the enzyme to an apparent homogeneity of a protein band at 110–112 kDa is achieved by detergent/high salt extraction from Triton X-100 insoluble pellets (Hayashi and Yamazaki, 1991; Koch, 1991; Margulis et al., 1993). Association with cytoskeletal structures was also demonstrated by direct interaction studies showing co-immunoprecipitation of ROS-GC1 and tubulin (Schrem et al., 1999) and binding of actin (Hallett et al., 1996) to the cyclase (Figure 4 of GC signaling complex). Based on translocation studies on mice lacking ROS-GC1 (GC-E^−/−^) it was suggested that ROS-GC1 is crucial for the intracellular trafficking of peripheral membrane proteins (Baehr et al., 2007; Karan et al., 2010). A co-immunoprecipitation study showed further that ROS-GC1 and the α–subunit of transducin form a complex, which might regulate the light-dependent translocation of transducin (Rosenzweig et al., 2009).

Both GCs are regulated by Ca^2+^-sensor proteins (Koch and Stryer, 1988) named GC-activating proteins (GCAP) that detect changes in cytoplasmic Ca^2+^ via their EF-hand Ca^2+^-binding motifs (Palczewski et al., 1994; Dizhoor et al., 1995; Frins et al., 1996). Guanylate cyclase-activating proteins (GCAPs) interact with the target GC at low and high Ca^2+^-concentration, although the binding affinity is not very high. Apparent affinity constants are in the lower micromolar to submicromolar range, which would allow transitory and flexible complex formation (Hwang and Koch, 2002; Peshenko et al., 2004, 2011a). Binding or dissociation of Ca^2+^ triggers conformational changes in GCAPs causing probably a rearrangement of the whole complex to increase or decrease GC activities (Sokal et al., 1999a; Hwang et al., 2001; Lim et al., 2009; Sulmann et al., 2014; Marino et al., 2015), which are high at low Ca^2+^ (and having Mg^2+^-bound in exchange for Ca^2+^; Peshenko and Dizhoor, 2006) and are low or even suppressed at saturating Ca^2+^-concentration. GCAPs are expressed in different isoforms from two or three in mammals to six or eight in teleost fish (Imanishi et al., 2004; Rätscho et al., 2009; Scholten and Koch, 2011; Fries et al., 2012). Expression of two or more isoforms in one cell type has raised questions as to the physiological meaning. Detailed biochemical characterization of GCAP properties in combination with the analysis of transgenic mice showed that GCAPs differ in their Ca^2+^-sensitivity, Ca^2+^-binding properties and target regulatory features (Mendez et al., 2001; Hwang et al., 2003; Peshenko et al., 2011b). These results led to a concept of GCAPs activating the target in a sequential order depending on the actual cytoplasmic Ca^2+^-concentration in the rod or cone cell (Hwang et al., 2003; Koch, 2006; Makino et al., 2012; Koch and Dell’Orco, 2013). For example, mammalian GCAP1 is active at higher Ca^2+^-concentration than GCAP2. This means in physiological terms that after a single flash of light, when Ca^2+^-decreases in the cell, GCAP1 will first loose its bound Ca^2+^ and turn into an activator before GCAP2 would step into this Ca^2+^-feedback loop. Electrophysiological recordings and computational modeling of photoresponses have further supported a Ca^2+^-relay or recruitment model of GCAP action (Koch and Dell’Orco, 2013; Wen et al., 2014). Based on differences in spatial-temporal expression profiles (Rätscho et al., 2009), Ca^2+^-sensitive target regulation and Ca^2+^-binding of multiple GCAP isoforms (Scholten and Koch, 2011; Sulmann et al., 2015) a similar concept has been proposed for the operation of these Ca^2+^-sensors in zebrafish rod and cone cells (Koch, 2013).

**Figure 5:**
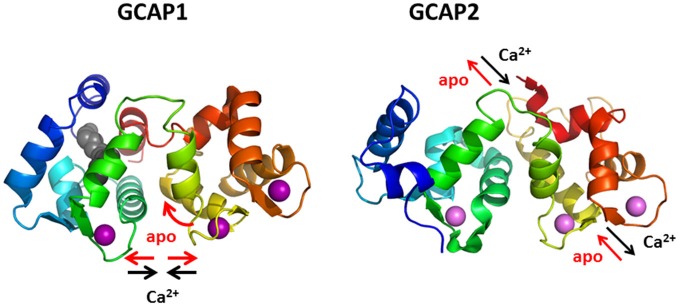
**Protein dynamics of GCAP1 and GCAP2.** Binding and dissociation of Ca^2+^ (violet spheres) triggers different conformational changes and movements of secondary structural elements, a twisted accordion-like movement in GCAP1 and a piston-like movement of one α-helix (yellow) in GCAP2.

GCAPs share a high degree of amino acid sequence identity and structural homology in cases where the three-dimensional folding had been determined (Ames et al., 1999; Stephen et al., 2007). However, the protein dynamics of Ca^2+^-triggered conformational changes show significant differences between mammalian GCAP1 and GCAP2 on a nanosecond time scale. Fluorescence lifetime and anisotropy measurements of GCAP1 and GCAP2 that were site specifically labeled with the dye Alexa647 revealed different movements of secondary structural elements (Kollmann et al., 2012; Robin et al., 2015). In the case of GCAP1 these findings were further supported by molecular dynamics simulation showing that GCAP1 undergoes a twisted accordion-like movement upon changing Ca^2+^-concentration (Robin et al., 2015). GCAP2 in contrast responds to a change in Ca^2+^ by an up-and-down or piston-like movement of an α-helix between amino acid positions 111 and 131 (Kollmann et al., 2012; Figure 5). A main conclusion from these studies was that two structurally similar proteins can differ significantly in their conformational structural dynamics thereby providing structural framework for their different regulatory responses.

Mutations in the retinal degeneration 3 protein (RD3) correlate with type 12 of Leber congenital amaurosis causing a severe form of blindness at early age (Friedman et al., 2006; Preising et al., 2012). The RD3 protein binds to photoreceptor specific GCs 1 and 2 (ROS-GC1 and ROS-GC2) and both proteins are not detectable in rods and cones of RD3 deficient mice (Azadi et al., 2010). Studies using RD3 and ROS-GC1 transfected COS-7 cells and by delivering the RD3 gene into an RD3 deficient mice strain by subretinal injection (Molday et al., 2013) revealed that RD3 is necessary for the correct translocation and cellular localization of ROS-GC1 (Azadi et al., 2010; Zulliger et al., 2015). RD3 does not only bind to GC1, but can act as an allosteric modulator inhibiting ROS-GC1 activity (Peshenko et al., 2011b; Figure 4).

The multiple binding and regulatory processes of the GC signaling unit leave several issues unresolved. For example, published work concerning the binding sites in the GC are inconclusive, since direct interaction sites for GCAP1 and GCAP2 have been mapped to different intracellular regions in ROS-GC1 (Figure 4) including the juxtamembrane domain (JMD; Duda et al., 1999a; Lange et al., 1999), the kinase homology domain (KHD; Krylov and Hurley, 2001) and the cyclase catalytic domain (CCD; Sokal et al., 1999b; Duda et al., 2005). Findings were based on different experimental approaches like peptide competition using peptide libraries, site-directed mutagenesis, and crosslinking in combination with mass spectrometry and might reflect different protein complexes due to the transitory nature of the GCAP-GC interaction mode (see above). However, a more recent paper claims that GCAP1 and GCAP2 bind to overlapping binding sites in a mutually exclusive manner (Peshenko et al., 2015a). A further major unresolved issue in understanding the GC signaling complex is how conformational changes in allosteric regulators (GCAPs) are transmitted to the catalytic site to increase the GC enzymatic turnover rate at least by a factor of 10. A critical role in sensing these conformational changes seems to play the dimerization domain (DD) of the cyclase. This domain forms a coiled—coil structure that is disrupted or in some cases even tightened in a number of retinal disease related mutations causing defects in the Ca^2+^-dependent control of GC activity (Duda et al., 1999b, 2000; Ramamurthy et al., 2001; Zägel et al., 2013). Very recently experimental evidence was presented showing that the DDis part of the GCAP binding interface (Peshenko et al., 2015b).

The differential regulation of GCs is not limited to GCAPs, but includes other Ca^2+^-sensor proteins as well. Neurocalcin δ (Kumar et al., 1999; Venkataraman et al., 2008) and S100B (Duda et al., 2002) target to binding sites in ROS-GC1 that are localized in the CCD or the C-terminal extension (CTE). Both Ca^2+^-sensors regulate the activity of ROS-GC1 in a Ca^2+^-dependent manner, but opposite to the GCAP mode indicating that these operation modes do not participate in outer segment physiology (Venkataraman et al., 2003), but are rather localized in other photoreceptor cell compartments, for example in modulating the signal transmission to cone ON-bipolar cells (Wen et al., 2012).

## Protein Assembly Providing a Structural Link Between Plasma and Disc Membrane

CNG channels are heterotetrameric proteins consisting, in rod and cone cells, of three α–subunits and one β–subunit (Weitz et al., 2002; Zheng et al., 2002). The subunits however are specific for each cell type and are designated CNGA1 and CNGB1 for the rod channel and CNGA3 and CNGB3 for the cone channel. The β–subunit CNGB1 of the rod channel has a unique structure, since it has an extended NH_2_-terminal part containing four proline-rich repeats and a glutamic acid rich part, which is therefore named glutamic acid rich protein (GARP) part (Sugimoto et al., 1991; Körschen et al., 1995; Colville and Molday, 1996). GARP is also expressed as two soluble protein variants (GARP1 and GARP2). Interaction of the GARP part of CNGB1 with disc membrane proteins at the rim region of the discs has been shown for the retina specific ABC-binding cassette (ABCR) transporter (Körschen et al., 1999) and for the peripherin-2/ROM-1 complex (Poetsch et al., 2001; Becirovic et al., 2014). The CNG channel interacts further in the plasma membrane with the Na^+^/Ca^2+^, K^+^-exchanger (Schwarzer et al., 2000; Kang et al., 2003). The oligomeric protein assembly around the CNG channel constitutes a highly organized microdomain in the rod cell consistent with a role of GARPs in maintaining structural integrity (Körschen et al., 1999; Poetsch et al., 2001; Zhang et al., 2009). The physiological role of different GARP variants however has not been fully explored and is still under discussion. For example, the soluble GARP variant GARP2 binds PDE6 with high affinity thereby inhibiting its basal activity (Pentia et al., 2006); it further controls the open probability of the CNG channel by acting as a gate keeper (Michalakis et al., 2011). These two mechanisms would lead to a reduction in dark noise (Pentia et al., 2006; Michalakis et al., 2011). Overexpressing GARP2 has an effect on the phototransduction gain as derived from fitting the rising phase of the electroretinogram a-wave response (Sarfare et al., 2014). The same study showed also a slower shutoff of the photoresponse. Reconciliation of physiological recordings with functional studies of isolated GARP proteins is hampered by their “intrinsically unfolded nature”, which is known from biochemical and structural analysis of GARP forms (Batra-Safferling et al., 2006). This property however, would make them suitable as flexible tethering proteins linking the CNG channel/exchanger complex with the rim region of the disc membrane.

## Concluding Remarks

Signaling proteins in photoreceptor cells form building blocks and signaling modules involving the disc membranes as platforms. These units might preferably exist of transitory protein complexes that are determined by the affinities of their binding partners and their lateral and longitudinal diffusion. This flexible regulatory network of proteins therefore creates the heterogeneous distribution of signaling units in the outer segment under conditions of a crowded intracellular environment. It will be a future challenge to investigate how the exchange, flow and mutual interaction of key factors in signaling units determines the preciseness of visual phototransduction and adaptation.

## Author Contributions

KWK developed the concept of the manuscript, wrote the first draft and approved the final version. DDO added to all parts of the manuscript and approved the final version.

## Conflict of Interest Statement

The authors declare that the research was conducted in the absence of any commercial or financial relationships that could be construed as a potential conflict of interest.
